# Noninvasive Home Mechanical Ventilation for Stable Hypercapnic COPD: A Clinical Respiratory Review from Canadian Perspectives

**DOI:** 10.1155/2023/8691539

**Published:** 2023-10-03

**Authors:** Rachel Jen, Colin Ellis, Marta Kaminska, Jeremy Road, Najib Ayas

**Affiliations:** ^1^Department of Medicine, Division of Respiratory Medicine, University of British Columbia, Vancouver, Canada; ^2^Department of Medicine, Division of Respirology, Critical Care and Sleep Medicine, University of Saskatchewan, Saskatoon, Canada; ^3^Respiratory Division and Sleep Laboratory, McGill University Health Centre, Montreal, Canada; ^4^Respiratory Epidemiology and Clinical Research Unit, Research Institute of the McGill University Health Centre, Montreal, Canada

## Abstract

Acute short-term noninvasive ventilation (NIV) for hypercapnic respiratory failure in chronic obstructive pulmonary disease (COPD) has well-established benefits; however, the role of long-term home NIV remains controversial. In the past decade, studies utilizing aggressive NIV settings to maximally reduce carbon dioxide levels (PaCO_2_) have resulted in several positive clinical trials and led to updated guidelines on home NIV for stable hypercapnic COPD patients. This clinical respiratory review discusses the high-intensity NIV approach, summarizes recent key trials and guidelines pertaining to home NIV in COPD, and considers key clinical questions for future research and application in the Canadian context. With recent evidence and Canadian Thoracic Society (CTS) guidelines supporting the use of NIV in carefully selected COPD patients with persistent daytime hypercapnia, we believe it is time to reconsider our approach.

## 1. Introduction

Chronic obstructive pulmonary disease (COPD) is the third leading cause of death globally [1], and moderate-to-severe COPD poses a considerable economic burden for healthcare providers globally [2]. 11.2% Canadians are estimated to have COPD [3], with 1.2% having severe airflow obstruction [4]. Furthermore, approximately 25% of moderate-to-severe COPD patients have a serum carbon dioxide level (PaCO_2_) above 45 mmHg, while 9% have a level greater than 50 mmHg [5]. We, thus, estimate that there are 7,000 Canadians aged 35–79 years with severe COPD and hypercapnia, with 2,500 having PaCO_2_ chronically above 50 mmHg [4].

Home mechanical ventilation (HMV) improves outcomes in hypercapnic patients due to restrictive lung diseases and neuromuscular diseases [6]. Home ventilation programs across Canada have provided this support for the past three decades following the 2011 Canadian HMV guideline, which did not endorse routine prescription of home NIV for COPD patients [7]. The benefits of HMV with noninvasive ventilation (NIV) in stable hypercapnic COPD patients only became evident in recent years as per updated guidelines by the European Respiratory Society (ERS) [8], American Thoracic Society (ATS) [9], and Canadian Thoracic Society (CTS) [10]. However, there are several practical limitations in implementing this therapy in Canada. One of them being that data come largely from Europe where the previous HMV trials were conducted [11, 12], where NIV was initiated in‐hospital over multiple days of adaptation; this is not practical in North America, particularly now in the COVID-19 pandemic context. Furthermore, varied insurance coverage for NIV in COPD posed significant challenges in North America. Hence, a technical expert panel in the United States of America (USA) has issued recommendations for policy changes to promote access to NIV therapy among COPD patients [13].

In addition, the NIV settings in previous NIV trials in COPD patients with chronic stable hypercapnia varied significantly. Those recent trials that showed clinical benefits used either high intensity (HI, high inspiratory pressure as well as mandatory respiratory rates higher than spontaneous breathing rates) or high pressure (e.g., starting inspiratory pressure of 18 cm H_2_O and aim to achieve IPAP more than 25 cm H_2_O) with the goal to substantially reduce levels of hypercapnia [11, 12]. These settings are much higher than those typically used in North America. Recent European trials have demonstrated that outpatient initiation and management of NIV is noninferior to in‐hospital initiation [14, 15]. In the Netherlands, NIV care has been successfully delivered in outpatient settings, and the NIV care delivery model in the Netherlands provides many useful insights for developing NIV care policy in Canada [16]. There are similarities (e.g., universal healthcare) and significant differences (e.g., geography in relation to specialized NIV centres) between Dutch and Canadian healthcare. Development of local expertise to NIV titration and monitoring at home in stable COPD patients across Canada is needed to implement this form of NIV effectively and safely.

Lastly, prior randomized controlled trials (RCTs) have generally excluded patients with obstructive sleep apnea (OSA), severe congestive heart failure, older age (>75 years), and elevated body mass index (BMI) except one [8]. In observational studies, ventilated COPD patients who were obese had better survival than nonobese patients, which might be an indicator of treatment of concomitant OSA and/or OHS as a contributor to hypercapnia [17]. However, the strict inclusion/exclusion criteria for prior RCTs may decrease the general applicability of NIV in North America. Anecdotally, an increased prevalence of obesity is observed in end-stage COPD patients in North America, in comparison to those in Europe [18]. We believe that obesity should not exclude COPD patients with chronic stable hypercapnia from NIV therapy. Rather, this should be managed with higher expiratory positive airway pressure (EPAP). However, this has not been evaluated by any RCT yet. This review will examine recent evidence and summarize practical consideration for clinicians when considering NIV in COPD patients with persistent daytime hypercapnia.

## 2. Methods

PubMed and Medline were searched for articles on long-term noninvasive ventilation in COPD up to August 1, 2022. Search terms included “COPD,” “respiration, artificial” subheading “noninvasive ventilation,” and “home mechanical ventilation.” Recent and relevant trials, review articles, and meta-analyses were prioritized. References were reviewed to ensure important research articles were not excluded.

## 3. Physiologic Benefits of NIV

Noninvasive positive pressure ventilation (NIV) works to counteract multiple pathophysiological processes, including respiratory muscle fatigue and dysfunction, altered pulmonary mechanics, and V/Q mismatch. NIV reduces diaphragm's effort, increases ventilation, reduces PCO_2_ due to increased ventilation, improves respiratory centre chemosensitivity, and reduces intercostal and diaphragm activities [19, 20].

All three recently published guidelines including ERS [8], ATS [9], and CTS [10] recommended considering initiation of home NIV in stable hypercapnic COPD patients to improve survival, albeit with low levels of certainty due to very limited data. CTS guidelines further favor high-intensity NIV (HI-NIV) instead of low-intensity NIV (LI-NIV).

## 4. “High-Intensity”/“High-Pressure” vs. “Low-Intensity” NIV

Despite the absence of standardized categories, NIV has been conceptualized into high intensity (HI)/high pressure (HP) and low intensity (LI). High-intensity/high-pressure NIV (HI/HP-NIV) involves progressively increasing the inspiratory positive airway pressure (IPAP) to the highest tolerated pressure up to 30 cm H_2_O in an attempt to maximally decrease PaCO_2_ [8]. Low-intensity settings (LI-NIV) use a low pressure and low backup rate. A further distinction has been made by some authors between high intensity (HI-NIV) and high pressure (HP-NIV). HI-NIV utilizes a high backup rate (BR), often set to within 2 breaths of the patient's own breathing rate to minimize patient triggering, in addition to a high-pressure NIV setting. The IPAP threshold between high- and low-pressure NIV has not been formally defined, although ≥18 cm H_2_O has been suggested [19].

A randomized crossover trial by Dreher et al. comparing HI-NIV (mean IPAP 28.6 cm H_2_O) vs. LI-NIV (mean IPAP 14.6 cm H_2_O) found several benefits of the HI-NIV strategy, including lower nocturnal PaCO_2_ (9.2 cm H_2_O lower, *p* = 0.001), improved dyspnea, and increased 6 minute walking distance (6MWD) [21]. Quality of life (QOL) was significantly improved above baseline only with HI-NIV [21]. With ventilation measured using a pneumotachograph, high-intensity settings resulted in higher volume delivered per breath (on average 325 mL), though a substantial portion was lost to leak, as the increase in the tidal volume was only 96 mL on average, HI-NIV vs. LI-NIV. Of note, initiation of HI-NIV took approximately 2.5 hospital-admission days longer than LI-NIV (mean time to titrate 4 vs. 1.6 days) [21].

It is unclear whether a high BR is necessary to achieve the benefits of HI-NIV. A randomized crossover trial including 12 patients by Murphy et al. compared HI-NIV vs. HP-NIV strategies [22]. The BR was set at 2 breaths below the patient's resting spontaneous breathing rate in the HI-NIV group and 6 breaths/minute in the HP-NIV group. They found no difference in mean ventilator usage, gas exchange, or sleep quality [22]. The results from this trial guided the rationale for using HP-NIV in the landmark HOT-HMV trial (home oxygen therapy versus home mechanical ventilation in COPD) [11].

The HOT-HMV trial by Murphy et al. randomized 116 patients with both hypercapnia (PaCO_2_ > 53 mmHg) and hypoxemia (PaO_2_ < 55 mmHg) following a recent acute hypercapnic exacerbation. Participants were randomized to receive both home oxygen therapy (HOT) and home mechanical ventilation (HMV-HP-NIV) (*n* = 57) versus HOT alone (*n* = 59). HOT-HMV significantly decreased the readmission or death (study composite endpoint) when compared to HOT alone: the median time to readmission or death in subjects in the HOT-HMV arm was 4.3 months compared to 1.4 months in the HOT arm. The endpoint was driven by a reduction in the COPD exacerbation rate (3.8 exacerbations per year in the HOT-HMV arm vs. 5.1 exacerbations per year in the HOT arm). There was no significant difference in the 12-month mortality rate between the groups (28% in HOT-HMV vs. 32% in HOT).

However, in another European RCT of stable patients with severe COPD and hypercapnia who had not had recent exacerbations, HI-NIV led to a substantial mortality reduction [12]. Kohnlein et al. randomized 195 patients with severe stable COPD and PaCO_2_ ≥ 52 mmHg to HI-NIV vs. medical therapy [12]. HI-NIV was utilized, with a goal reduction of baseline PaCO_2_ by 20% or to a value of ≤48 mmHg. The mortality rate in one year was significantly reduced in the HI-NIV group (12 vs. 33%; *p* = 0.0004), and quality of life was significantly improved in the HI-NIV group. The results of Kohnlein's and the HOT-HMV trials led to the recent recommendations by ERS, ATS, and CTS guidelines after many years of controversy regarding NIV therapy in COPD [8–10]. The most recent systemic review on home NIV in COPD showed that NIV may reduce hospital admissions and improve quality of life, but there is still little evidence of a reduction in mortality [23].

## 5. Who and When to Use HI/HP-NIV in COPD

Appropriate patient selection is critical for long-term NIV to improve patient outcomes. Recent guidelines' recommendations are shaped by the results of three recent RCTs, which are summarized in Table 1. Even though the required level of baseline hypercapnia in stable COPD patients in order to benefit from NIV is not clear, CTS guidelines suggested only considering NIV in patients with persistent daytime PaCO_2_ ≥ 52 mmHg, which is close to the cutoff level for the HOT-HMV trial, as greater benefit appears to occur in patients with higher levels of baseline hypercapnia [10]. On the other hand, ATS guidelines chose daytime PaCO_2_ ≥ 45 mmHg as the cutoff level for NIV initiation for stable COPD patients [9].

In addition, ERS/ATS/CTS guidelines emphasized to only start long-term NIV in COPD patients with “stable hypercapnia,” not immediately after COPD exacerbation [8–10]. This recommendation was drawn from the contradictory results of two large RCTs (HOT-HMV trial and RESCUE trial) that investigated the clinical efficacy of NIV following a life-threatening exacerbation of COPD requiring acute NIV in‐hospital. In the RESCUE trial, Struik et al. randomized 201 patients to HI-NIV vs. no NIV if they remained hypercapnic for >48 hours after discontinuation of acute NIV during hospitalization for an acute exacerbation of COPD (AECOPD) [24]. There was no significant improvement in the primary outcome of readmission to hospital for any respiratory cause or death within 12 months. Of note, PaCO_2_ at study termination was almost identical in the two groups (48 vs. 49.5 mmHg, NIV vs. no NIV, respectively), and 26% of patients in the control group became eucapnic at 3 months [24]. The inclusion of patients who ultimately did not have chronic hypercapnia may be one main reason for the negative results of the study, despite the use of HI-NIV. Like the RESCUE trial, HOT-HMV was a “post‐exacerbation” trial with a key difference in timing of enrollment [11]. In HOT-HMV, patients were enrolled if they had a PaCO_2_ > 53 mmHg 2–4 weeks after resolution of AECOPD. The HOT-HMV trial has shown NIV significantly reduced COPD exacerbation. Thus, ATS/CTS guidelines suggested not initiating long-term NIV during admission, favoring reassessment for NIV in 2–4 weeks after discharge [9, 10]. A recent cohort study by Frazier has indicated that early initiation of NIV after diagnosis of hypercapnia and COPD may reduce mortality among COPD patients [25]. This may further support the recommendation by ERS regarding NIV initiation; reassessment after COPD exacerbation could be considered but was not necessary. It should be left up to the discretion of the treating physician [8].

## 6. OSA-COPD Overlap

As all HI-NIV RCT trials for COPD with stable hypercapnia have been conducted in Europe, it is unclear whether HI-NIV can be effectively applied in North America. In North America, there has only been one single-centre retrospective study from Galli that examined the impact of HI-NIV in clinical practice in 166 participants with chronic hypercapnia (PaCO_2_ > 45 mm·Hg) admitted to the intensive care unit for acute exacerbation of COPD [26]. HI-NIV was associated with a reduction in‐hospital readmissions (40% vs. 75%; *p* = 0.002) and a nonsignificantly decreased mortality rate (10% vs. 19%; *p* = 0.13) at 6 months. However, 47% of the NIV group had a history of obstructive sleep apnea (OSA)/obesity hypoventilation syndrome (OHS), and the average BMI was 33.7 kg/m^2^. This is very different from European HI-NIV studies as the average BMI was 24.7 and 21.6 kg/m^2^ in Struik's study and the HOT-HMV trial, respectively [11, 24]. For Galli's study, it is unclear how much treatment benefits may be explained by treating OSA/OHS.

With some exceptions, RCTs have generally excluded patients with obstructive sleep apnea (OSA) and the elevated body mass index (BMI) [11, 12, 24]. In observational studies, ventilated COPD patients who were obese had better survival than nonobese patients, which might be an indicator of concomitant OSA and/or OHS [17].

The coexistence of COPD and OSA in the same patient, termed overlap syndrome (OVS), affects 1% of the North American population (an estimated 300,000 in Canada alone) [27]. Patients with OVS suffer the combined detrimental effects of COPD and OSA; these result in substantial health consequences including increased mortality [28], worse nocturnal desaturation [29], worse cardiopulmonary hemodynamics (e.g., pulmonary hypertension and right ventricular dysfunction) [30, 31], and increased risk of chronic hypercapnia [29, 32]. Patients with untreated overlap syndrome may have worse survival than those treated with CPAP [28]. Recognizing the importance of coexisting OSA in COPD patients, ATS guidelines suggest chronic stable hypercapnic COPD patients undergo screening for obstructive sleep apnea before initiation of long-term NIV [9, 33]. We believe that OSA should not be considered a contraindication for NIV in appropriately selected individuals, though treatment with CPAP alone may be considered if OHS as opposed to COPD is felt to be the main driver of hypercapnia.

## 7. How to Use NIV

HI-NIV in European trials was initiated in hospitals, over several days for all HI/HP-NIV RCT trials for COPD with stable hypercapnia [11, 12, 24]. Titration was monitored with nocturnal transcutaneous carbon dioxide (TcCO_2_) monitoring or morning arterial blood gases (ABGs), but without formal polysomnography [11, 12, 24]. It is currently prohibitively expensive to follow the European inpatient initiation and titration protocol of HI/HP-NIV in most North American centres. Therefore, alternatives for NIV initiation strategies need to be evaluated in Canada. With advancement of telemedicine, a recent RCT by Duiverman et al. found no difference in CO_2_ reduction or QOL at 6 months while comparing home and telemedicine versus in‐hospital initiation of NIV [15]. Adherence was better in the home-initiation group. This suggests home initiation of NIV can be successfully performed for COPD patients, but local expertise in NIV is needed for a successful NIV program. An approach in the Netherlands to manage NIV in selected centres with NIV expertise may provide useful insights but probably needs to be modified due to the geographical and funding model differences between the Netherlands and Canada [34].

When considering how to initiate NIV, titration polysomnography (PSG) is often considered in Canada. However, it is well recognized that optimal settings of HI/HP-NIV are difficult to achieve overnight without several days of gradual patient adaptation and adjustments. ATS guidelines suggest not using PSG to titrate NIV as it can increase therapy cost, potentially delay therapy, and may cause harm (e.g., glottic closure) with aggressive titration during a single night [9].

A new NIV mode has also been considered by many as an option to simplify outpatient NIV titration for COPD. Multiple trials have compared volume-assured pressure support (VAPS) to standard pressure-targeted modes [35–38]. The benefit of VAPS is stability of tidal volume delivered by the device, together with the benefits of pressure-targeted ventilation, mainly leak compensation, which is relevant due to the large leak often associated with higher IPAP used in HI-NIV. However, VAPS modes may not be effective when large leaks are present. Both ERS and CTS guidelines recommend against VAPS over standard pressure-preset NIV as there has been little evidence to date that VAPS is superior to standard pressure-targeted treatment in terms of ventilation, sleep quality, or quality of life [8, 10]. Using Medicare data between 2016 and 2020 in the USA, a cohort study of COPD patients with stable hypercapnia and treatment with NIV revealed the devices used in the real world may be quite different from the guidelines' recommendations: 6.6% received bilevel positive airway pressure (BPAP) and 92% received home ventilators, which are more expensive with additional features/modes for ventilatory support [25]. As discussed in the Technical Expert Panel Report, the hardware and software of these devices have gone through major advancement. More research will be needed in this area to identify the optimal device/mode for COPD patients with hypercapnia.

Lastly, ERS/ATS/CTS guidelines suggest that the aim of NIV therapy should be reduction or normalization of PaCO_2_ to achieve control of nocturnal hypoventilation [8–10]. Thus, regular monitoring and optimization of NIV settings over time is an integral part of the NIV treatment plan.

## 8. Canadian Perspective

There is strong support for NIV and home ventilation for hypercapnic patients with neuromuscular and chest wall diseases; there are well-established benefits of reduced need for hospitalization, improved quality of life, and prolonged survival [7]. Home ventilation programs across Canada have provided this support for the past three decades.

The Canadian home mechanical ventilation guideline from 2011 did not endorse routine prescription of home NIV for COPD [7]. Overall, COPD has been an uncommon indication for home NIV in Canada, but there is significant regional variability [39]. Povitz et al. found that COPD was one of the leading indications for HMV in Ontario, accounting for 18.8% of HMV patients from 2000 to 2012 [40]. Since 2021 CTS guidelines [10] recommended consideration of NIV therapy in selected COPD patients with persistent daytime hypercapnia (PaCO_2_ ≥ 52 mm·Hg), we should see increased adaption of NIV in this population. However, the HI/HP-NIV settings recommended by guidelines [8–10] are more aggressive than what has traditionally been used, requiring education and support for healthcare providers. Development of local expertise to guide HP/HI-NIV titration and monitoring of PaCO_2_ noninvasively in stable COPD patients are needed to implement NIV effectively and safely.

Given the potential volume of COPD patients who could qualify for home NIV, the implementation in Canada requires careful evaluation by stakeholders involved in home ventilation programs. A systematic review showing the cost-effectiveness of home NIV remains uncertain in 2015, but the findings were sensitive to emergent data [41]. A recent report using Medicare data showed reduction in Medicare expenditures with early initiation of NIV in COPD patients with chronic hypercapnia [25]. This is very encouraging, and more data will be helpful in the implementation of NIV therapy as per guideline recommendations in Canada [25].

## 9. Future Research Recommendations

The quality of evidence supporting the benefits of home NIV for COPD is low to moderate, with the best evidence attributable to two recent RCTs that employed HI/HP-NIV treatment strategies [11, 12]. Although there is promising evidence for the benefit of HI/HP-NIV for mortality and hospitalizations, further research will play a major role in improving the confidence in these findings and in helping to guide the policy. Patient-centred outcomes should be evaluated in a standardized fashion, especially in the context of HI/HP-NIV, including quality of life, sleep quality, and exercise capacity.

The use of HI/HP-NIV in patients with COPD and comorbidities such as OSA and heart failure merits further research. More evidence guiding optimal NIV settings (including BR), use of VAPS modes and other novel modes [42], outpatient procedures for initiation of NIV, and targets for optimal titration should be further explored.

## 10. Conclusion

There is promising evidence that NIV is effective for patients with hypercapnic COPD. Careful patient selection and application of high-intensity/high-pressure NIV with the goal of normalizing or achieving maximal reduction in PaCO_2_ appear to be important for improving outcomes. With guidelines cautiously suggesting NIV in selected COPD patients, we need to incorporate the evidence into our policy to establish local expertise and practical approaches in Canada.

## Figures and Tables

**Table 1:** Landmark RCTs of high-intensity NIV.

	Kohnlein [12]	Struik [24]	Murphy [11]
*N*	195	201	116
Age	62 vs. 64	64 vs. 63.5	66.4 vs. 67.1
Post-AECOPD	No	Yes	Yes
Follow-up	12 months	12 months	12 months
BMI (kg/m^2^)	24.8 vs. 24.5	24.6 vs. 24.8	21.5 vs. 22.2
FEV_1_ (% predicted)	26 vs. 27.5	25.6 vs. 25.7	24 vs. 22.9
Inclusion minimal PaCO_2_ criteria (mmHg)	51.9	45	53
Baseline PaCO_2_ (mmHg)	58.5 vs. 57.8	59.3 vs. 57.8	59
Final IPAP/EPAP (cm H_2_O)	21.6/4.8	19.2/4.8	24/4
Final backup rate (breaths/minute)	14	15	14
Adherence (hour/night)	5.9	6.9	7.6
Outcome	LTH-NIV improved 1-year all-cause mortality	LTH-NIV did not improve time to readmission or death	LTH-NIV improved time to readmission or death

^*∗*^Compared values are intervention vs. control. Data are shown as the mean unless otherwise indicated.
